# A case report on radiation-induced angiosarcoma of breast post skin-sparing mastectomy and reconstruction with transverse rectus abdominal muscle

**DOI:** 10.3332/ecancer.2014.402

**Published:** 2014-02-17

**Authors:** Adil Aljarrah, Claude Nos, Krishna B Clough, Marie Aude Lefrere-Belda, Fabrice Lecuru

**Affiliations:** 1 Department of Gynecologic and Oncologic Surgery, Hôpital Européen Georges Pompidou,12 Rue Leblanc, 75015 Paris, France; 2 Institut du Sein, Paris Breast Center, 7 Avenue Bugeaud, 75 016 Paris, France; 3 Breast Unit, Department Of Surgery, Sultan Qaboos university Hospital, P. O. Box 38 PC 112, Muscat, Sultanate of Oman

**Keywords:** breast carcinoma-angiosarcoma-radiotherapy, TRAM

## Abstract

Radiation-induced angiosarcomas (RIA) are rare tumours that can affect breast cancer patients following treatment with breast conservative surgery and radiotherapy. Their diagnosis is often delayed because of their benign appearance and the difficulty in differentiation from radiation-induced skin changes. We report here a case of RIA which occurred seven years after radiotherapy to highlight awareness of the disease and the role of careful histological evaluation of these tumours.

## Introduction

Conservative breast surgery that is followed by radiotherapy is becoming more practised as a result of good screening programmes detecting early breast cancer. Radiation-induced angiosarcoma is a known complication of radiotherapy for breast cancer. Angiosarcomas of the breast are rare tumours of endovascular origin. Primary angiosarcomas account for only 0.04% of breast tumours and affect a younger age group of patients (20–40 years old) in comparison with adenocarcinomas. Primary angiosarcomas are extremely aggressive neoplasms. A review of 219 published cases reported a ten year survival rate of 17% [1]. Secondary angiosarcomas can occur as a result of chronic lymphoedematous arms after axillary dissection for breast cancer (Stewart–Treves syndrome) or at the site of irradiated skin, radiation-induced angiosarcomas (RIA); they also have poor prognosis [1]. There is often delay in the diagnosis of RIA despite the fact that most of these patients are under regular surveillance for recurrence of breast carcinoma [2, 3]. We report here a case of RIA to highlight awareness of the disease and the pitfalls to avoid in order to minimise delays in the diagnosis.

## Case report

**Figure 1.**
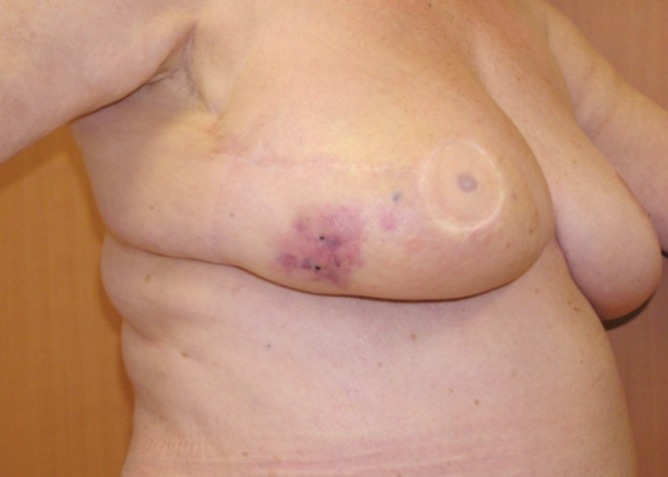
Radiation-induced angiosarcoma (right breast) occurring on irradiated skin in December 2007.

**Figure 2.**
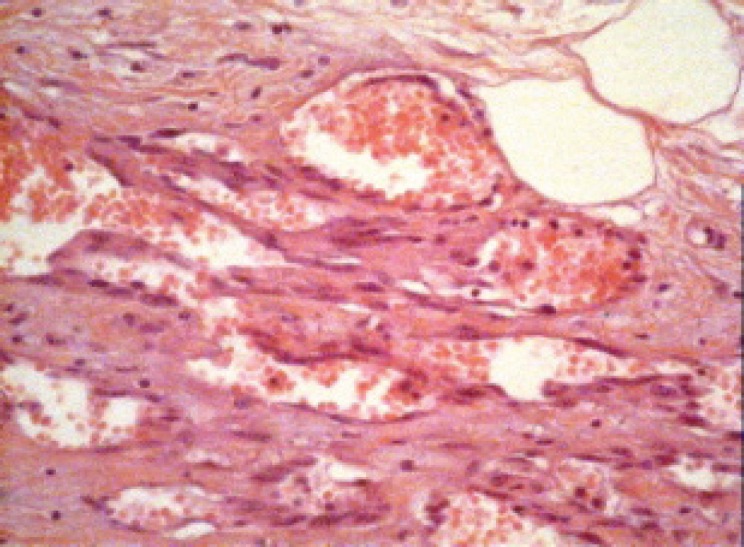
This high power view (×40 magnification) shows neoplastic vascular channels. These irregular channels interconnect and are lined by a single layer of highly atypical endothelial.

A 56-year-old patient was diagnosed with right breast ductal carcinoma in situ in December 2000. She underwent conservative breast surgery and axillary dissection with symmetrisation of the contralateral breast, as part of an oncoplastic procedure. Her postoperative recovery was eventful, and she commenced radiotherapy treatment in March 2001. She received 48 Gy in 26 fractions over 46 days, using paired glancing megavoltage fields. She did not receive any boost. Two years later, on a routine follow-up, she was found to have an abnormal right mammogram. A biopsy of the right breast lesion confirmed recurrence with invasive ductal carcinoma. She underwent skin-sparing right mastectomy and immediate breast reconstruction using transverse rectus abdominal muscle (TRAM) flap. She made an unremarkable recovery. In June 2007, that is, six years and three months after her radiotherapy, the patient noticed a purplish spot in the skin of the previously irradiated field of the right breast. She consulted her physician three months later as the spot had increased in size and was now about 3 × 3 cm. A mammogram and ultrasound were performed and showed an area of tissue oedema without any underlying tissue changes other than some cystic changes. The physician concluded that the patient had hypodermatitis secondary to radiotherapy with no evidence of recurrence. Six months after her initial symptom, the patient was reviewed by a surgeon as part of a routine annual follow-up. It was noted that the skin ‘spot’ was now 7 × 5 cm in size and was associated with indurations, thickness, and itching in the lower outer quadrant of the right breast (Figure 1). Two punch biopsies were performed which on histological examination showed dermal vascular changes with atypia but no mitosis or signs of malignancy. She was seen three months later by a dermatologist who requested expert review of the previous slides to exclude angiosarcoma. The histopathology review confirmed a diagnosis of angiosarcoma. Urgent computerised tomography scans of her chest, abdomen, and pelvis showed no evidence of metastasis. The patient subsequently underwent radical surgical treatment (excision of the skin, the flap, and axillary lymph nodes). The histopathological examination showed that an inflammatory area of 5 × 4 cm with angiosarcoma grade I, anti-CD 31, and CD 34, was positive; the lymph nodes were free of metastasis (Figure 2). She remained apparently well till January 2009 when she developed a second recurrence in the scar. She refused any further surgical intervention. After four courses of doxorubicin–ifosfamide and a course of sorafenib, the patient had complete clinical and histological remission [4, 5].

## Discussion

RIA was first reported in the literature in 1929 [6], and the first case of sarcoma after radiotherapy for breast cancer was reported by Warren and Sommer in 1936 [7]. The diagnostic criteria for RIA include a previous history of radiotherapy with a latency period of several years (five or more), development of sarcoma within a previous irradiated field, and a histology confirmation. RIA are characterised by their aggressive nature and most of them are high-grade tumours [2]. The prognosis is poor and local recurrence rates approach 70% after mastectomy. Survival depends on the sarcoma grade and the interval between diagnosis and treatment. A two-year disease-free survival ranges from 0 to 35% in different publications [1, 2]. It is difficult to analyse the relationship between the total radiation dose and the incidence of RIA, but a minimum total dose of 10 Gy in conventional doses per fraction appears necessary to result in RIA [8]. The incidence of RIA following radiotherapy to the breast varies from 0.05 to 0.2; the mean time was six years [8], similar to our case.

The presentation of RIA is often as a cutaneous or subcutaneous lesion, painless, flat or nodular, bluish or purplish similar to benign angiomas, small hematomas, or atypical telangiectasia [2]. The diagnosis can be delayed up to 8–12 months [3]. In our study, the delay was almost six months. Diagnosis is often difficult due to its rarity, benign appearance, and difficulty in differentiation from radiation-induced changes in the skin following radiotherapy. Preoperative diagnosis of angiosarcoma of the breast, by aspiration cytology and biopsy, is often difficult. Chen et al reported that the false negative rate of biopsy was 37% [9]. The difficulties in the clinical diagnosis of angiosarcoma of the breast cause delay in the diagnosis, and this results in poor prognosis [3]. However, even with early skin biopsy, delays can be encountered due to the difficulty in detecting the tumor in previously irradiated tissue or inadequate tissue for the biopsy [10]. A high index of suspicion, careful patient evaluation, and adequate biopsy tissue for pathological diagnosis are mandatory. Mammography is typically negative in these cases. The diagnosis can be confirmed only by histopathological studies as cytology is usually not conclusive [10]. Conservative surgical treatment regardless of margin involvement will expose the patient to early recurrences and metastatic spread [9–11].

Adjuvant chemotherapy in RIA is still not widely used and has shown disappointing results (new) [12]. Radiation therapy has been avoided in these secondary cases because of concerns about the toxicity of repeated treatment. However, recently some encouraging results have been achieved with the use of hyperfractionated radiotherapy (new) [11]. Metastases derived from mammary angiosarcomas have been reported in lung, skin, liver, bone, central nervous system, spleen, ovary, lymph nodes, and heart. Axillary lymph node metastases can occur and are only numbered three among the previously reported cases as the site of metastasis [11].

## Conclusion

RIA is rare, but with reduction of number of mastectomies in favour of conservative breast surgery which is followed by radiotherapy, the incidence might increase. Because RIA tends to occur years after radiotherapy, treating and following up physicians should be aware of the appearance of the disease and to perform large excisional biopsy for early diagnosis.
